# Effects of Extreme Temperature on Human Bronchial Epithelial Cells in 3D Printed Samples

**DOI:** 10.3390/bioengineering11121201

**Published:** 2024-11-28

**Authors:** Taieba Tuba Rahman, Nathan Wood, Zhijian Pei, Hongmin Qin

**Affiliations:** 1 Department of Industrial & Systems Engineering, Texas A&M University, College Station, TX 77843, USA; zjpei@tamu.edu; 2 Department of Biology, Texas A&M University, College Station, TX 77843, USA; woodn@tamu.edu (N.W.); hqin@tamu.edu (H.Q.)

**Keywords:** 3D printing, cell viability, extreme temperature, human bronchial epithelial cells, mitochondrial oxidative stress

## Abstract

This paper reports an experimental study on the effects of extreme temperature on human bronchial epithelial (HBE) cells encapsulated in 3D printed samples. Well plates of the 3D printed samples were exposed to three levels of temperature (37 °C, 45 °C, and 55 °C, respectively) for a duration of 10 min. Cells’ responses, specifically cell viability and oxidative stress, were quantified using Hoechst 33342, Sytox, and Mitosox stains, with intensity measurements obtained via a plate reader. In addition, cell viability was assessed through microscopic imaging of the 3D printed samples. Experimental results demonstrated that the temperature increase from 37 °C to 55 °C significantly reduced nuclear integrity as observed through Hoechst 33342 intensity, while increased Sytox intensity reflected a higher degree of cell death. Furthermore, cells exposed to 45 °C and 55 °C exhibited decreased cell viability and elevated mitochondrial oxidative stress. These findings offer valuable insights into the effects of extreme temperature on HBE cells, establishing a foundation for future research into how respiratory tissues respond to thermal stress. This research can potentially advance the knowledge regarding effects of heat exposure on the respiratory system.

## 1. Introduction

The standard core body temperature for humans is 37 °C. When core body temperature surpasses 37.5 °C, the condition is defined as hyperthermia [1,2]. Hyperthermia poses severe challenges to the human body’s ability to maintain homeostasis, a condition of internal balance that ensures metabolic stability as organisms adapt to external environmental changes [2,3,4]. Hyperthermia poses occupational health risks to professionals regularly exposed to extreme high temperatures. For example, fighter pilots routinely experience high temperatures during pre-flight duties and low-altitude missions, with cockpit temperatures exceeding 45 °C [5,6,7,8]. Hyperthermia can cause discomfort, along with dehydration, fatigue, and potentially more severe conditions such as heat stroke and heat cramps [9,10,11]. This can lead to declines in cognitive performance, such as impaired vigilance, slower reaction times, and poor decision-making [5,9]. 

Table 1 lists some reported studies on the effects of extreme temperature using human subjects [5,12], in vitro cellular models [13,14,15], and invertebrate models [16]. For instance, Zhou et al. investigated the physiological characteristics of pilots during simulated fighter cockpit environments. They found that the pulse rate, core temperature, mean skin temperature, and sweat amount of human subjects increased markedly with elevating temperature and relative humidity [5]. Some other studies, using in vitro cellular models, have investigated the effects of extreme temperature on mouse neural stem cells [13], human mesenchymal stem cells [14], and rats’ lung tissues [15]. However, the effects of extreme temperature on human bronchial epithelial (HBE) cells (crucial components of the lung) remain underexplored.

The lung is the most important organ for heat dissipation and oxygen exchange [15]. HBE cells form the first line of defense against inhaled pathogens and environmental pollutants. Investigating the effects of extreme temperature on HBE cells at the cellular level will provide insights into the cellular mechanisms of thermal injury, such as cellular membrane disruption, mitochondrial oxidative stress, cell death, and impaired cellular function. This study aims to fill the research gap by investigating the effects of extreme temperature on HBE cells.

## 2. Materials and Methods

**Figure 1:**
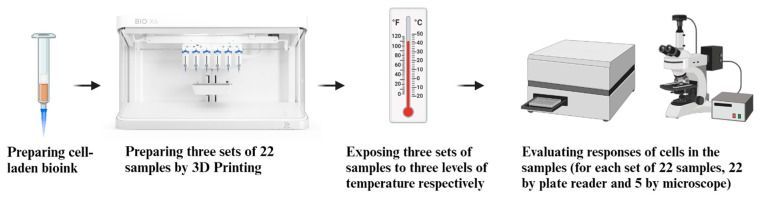
Overview of the experiment.

Figure 1 is an overview of the experiment.

### 2.1. Cell Culture

16HBE14o- human bronchial epithelial cells (Cat. No. SCC150), isolated from a 1-year-old male heart-lung patient, were purchased from Millipore Sigma. The purchased (cryopreserved) cells were cultured following the procedure described in an earlier paper [17]. 16HBE14o- cells in their sixth passage were used for this study. They were cultured in α-MEM medium (cell growth medium) (Sigma Cat. No. M2279, Millipore Sigma, Burlington, MA, USA) supplemented with both fetal bovine serum (10% *v*/*v*, Corning) and L-glutamine (2 mM, Corning, New York, NY, USA). They were cultured without any antibiotics or antimycotics until the initiation of experiments. When the cells reached 90% confluency, cell passaging was conducted following the procedure described in an earlier paper [17]. After cell passaging, the HBE cell concentration was approximately 6.35 × 10^5^ cells per milliliter of medium.

### 2.2. Preparation of Cell-Laden Bioink

Bioink was prepared with a 10% sodium alginate solution and neutralized collagen solution. The 10% (*w*/*v*) sodium alginate solution (from alginic acid sodium salt powder, product number: A1112, Sigma-Aldrich, Saint Louis, MO, USA) was prepared following the procedure described in an earlier paper [17]. The prepared sodium alginate solution in the beaker was sterilized via autoclave at 121 °C for 20 min and was then stored in a refrigerator at 4 °C. The collagen stock solution (TeloCol-10, type 1 bovine collagen, Advanced Biomatrix Carlsbad, CA, USA) had a concentration of 10 mg/mL. The collagen stock solution was neutralized following the procedure described in an earlier paper [17]. The collagen neutralization procedure was conducted in an ice bath. The ice bath was prepared by mixing crushed ice and water in a container, creating a stable cooling environment. It was used to keep solutions at a temperature close to 0 °C to prevent premature thermal gelation.

To prepare the bioink with a composition of 80% collagen and 20% alginate (or collagen–alginate 4:1 bioink), 1.6 mL of neutralized collagen solution was added into a sterile conical tube with 0.4 mL of 10% alginate solution. Throughout the preparation, both the 15 mL conical tube containing alginate solution and the 15 mL conical tube containing neutralized collagen solution were submerged in an ice bath. Approximately 50–70% of the conical tubes were submerged in the ice bath. The conical tube containing bioink was stored in the ice bath prior to the addition of cells to prevent premature thermal gelation.

To prepare cell-laden bioink, 1 mL of the HBE cells with medium (prepared by following the procedure described in Section 2.1) was added to the 2 mL of bioink, with an effective cell density of approximately 2.12 × 10^5^ cells per milliliter of bioink.

### 2.3. Preparation of Three Well Plates for 3D Printing

**Figure 2:**
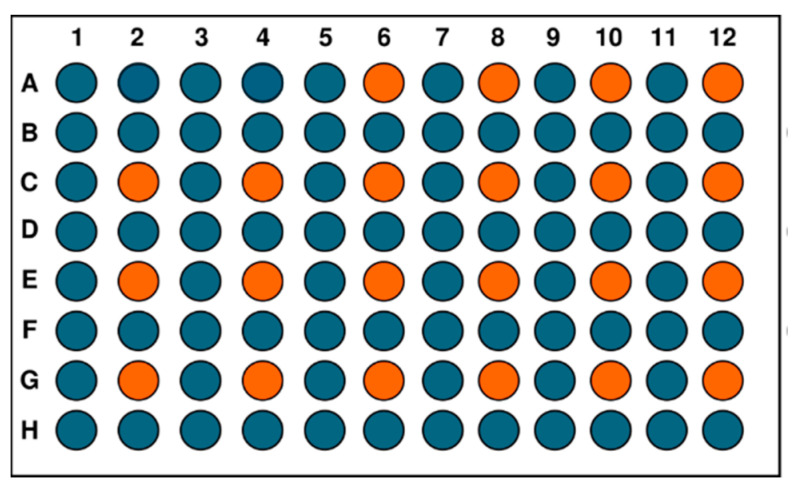
Designed layout of the 96-well plate; 76 wells (marked in blue color) were for 1X PBS and 22 wells (marked in orange color) were for 3D printing of samples.

Three individual 96-well plates, well plate 1, well plate 2, and well plate 3, were prepared for 3D printing. The three well-plates had the same designed layout, as illustrated in Figure 2. For each well plate, 74 wells (marked in blue color) were filled with 100 µL of 1X PBS and incubated for 24 h in the incubator at 37 °C with 5% CO_2_. The remaining 22 wells (marked in orange color) were kept for 3D printing of samples. The primary purpose of filling the 74 wells with 1X PBS was to maintain a humid environment within the well plate and prevent the printed samples from drying out.

### 2.4. 3D Printing of Samples on Well Plates

In this study, three sets of samples were prepared using 3D printing of cell-laden droplets. Well plate 1 was taken out from the incubator and put inside the bioprinter (Cell ink, BioX6, Gothenburg, Sweden). After samples were printed in the 22 wells of well plate 1, well plate 1 was taken out from the printer and put in a biosafety cabinet. Then, well plate 2 was taken out from the incubator and put inside the printer and samples were printed in its 22 wells. Afterwards, well plate 2 was taken out from the printer and put in the biosafety cabinet. Then, well plate 3 was taken out from the incubator and put inside the printer, and samples were printed in its 22 wells. Afterwards, well plate 3 was taken out from the printer and put in the biosafety cabinet.

**Figure 3:**
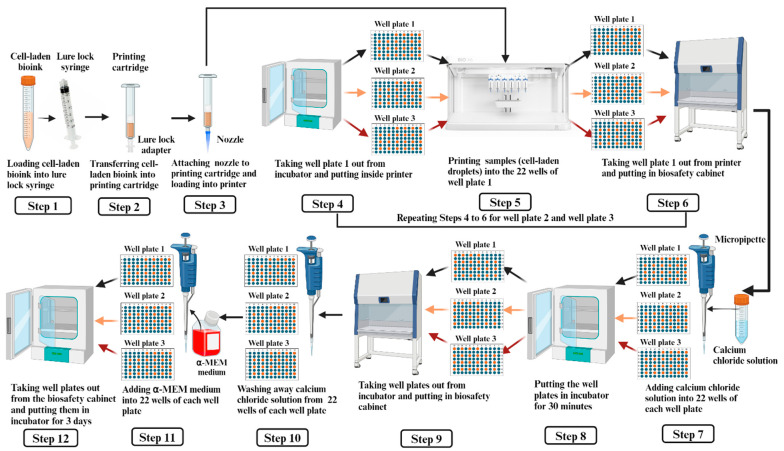
Steps for 3D printing of samples.

The steps for 3D printing of samples on each well plate are illustrated in Figure 3 and described below.

Step 1: Loading the cell-laden bioink (prepared by following the procedure described in Section 2.2) into a sterile lure lock syringe.

Step 2: Transferring the cell-laden bioink from the lure lock syringe to a 3 mL printing cartridge, using a lure lock adapter. As the printing cartridge did not have a pull-up handle, the lure lock syringe was used to pull the bioink from the conical tube.

Step 3: Attaching the 22 G (gauge) nozzle to the printing cartridge and loading the cartridge into the pneumatic printhead of the BioX6 3D printer (Cell ink, BioX6, Gothenburg, Sweden).

Step 4: Taking well plate 1 (prepared by following the procedure described in Section 2.3) out from the incubator and putting it inside the printer.

Step 5: At room temperature, printing samples (cell-laden droplets) into 22 wells of well plate 1 (one droplet in each well). The values of the printing parameters were an extrusion pressure of 10 KPa, extrusion time of 1.25 s, and extrusion height (the distance of the nozzle tip from the wells) of 0.20 mm. The extrusion height of 0.20 mm was selected to ensure precise deposition of cell-laden droplets while minimizing shear stress on cells during deposition, preserving cell viability and structural fidelity in printed samples.

Step 6: Taking well plate 1 out from the printer and putting it in the biosafety cabinet.

Steps 4 to 6 were repeated for well plate 2 and well plate 3.

Step 7: In the biosafety cabinet, using a micropipette, adding 100 µL of 50 mM calcium chloride solution to each of the 22 wells in each well plate, one well plate at a time, for ionic crosslinking of bioink.

Step 8: Putting the three well plates in a humidified incubator (at 37 °C with 5% CO_2_) for 30 min for thermal gelation of the bioink.

Step 9: Taking the three well plates out from the incubator and putting them in the biosafety cabinet.

Step 10: In the biosafety cabinet, using a micropipette, washing away calcium chloride solution from the 22 wells of each well plate, one well plate at a time.

Step 11: Using a micropipette, adding 100 µL of α-MEM medium to each of the 22 wells in each well plate, one well plate at a time.

Step 12: Taking the three well plates out from the biosafety cabinet and putting them in a humidified incubator (at 37 °C with 5% CO_2_) for 3 days.

### 2.5. Assessment of HBE Cells’ Responses

**Figure 4:**
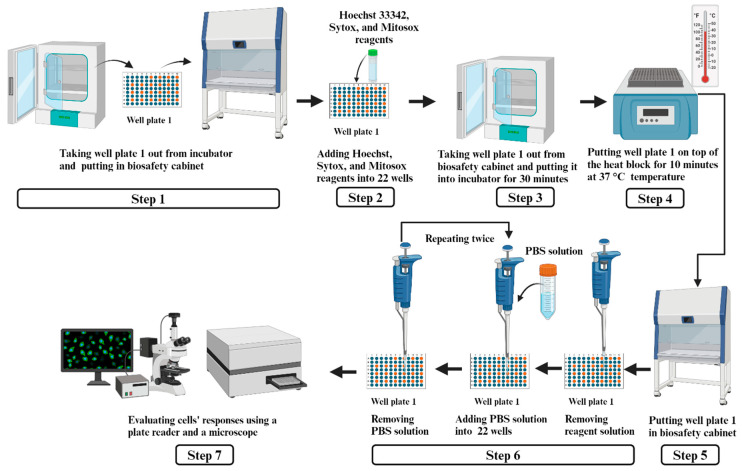
Assessment procedure of HBE cells’ responses.

The assessment procedure of cells’ responses for well plate 1 is described below and illustrated in Figure 4. These steps were repeated for well plate 2 and well plate 3. The only difference for assessing the three well plates was Step 4, with well plate 1 being treated at 37 °C, well plate 2 at 45 °C, and well plate 3 at 55 °C.

Step 1: Taking well plate 1 with 3D printed samples (prepared by following the procedure described in Section 2.4) out from the incubator and putting it in the biosafety cabinet.

Step 2: At room temperature, in the biosafety cabinet, adding 100 µL of combined reagents (Hoechst 33342, Sytox, and Mitosox) (Thermo Fisher Scientific, Waltham, MA, USA) into the 22 wells of well plate 1. The fluorescence stains (Hoechst 33342, Sytox, and Mitosox) were chosen for their high specificity and sensitivity (for DNA content, cell death, and mitochondrial ROS, respectively). The 30 min staining time ensured sufficient binding and signal generation without overexposure (overexposure could lead to nonspecific staining or signal degradation). Fluorescence stains and staining time were validated in preliminary experiments to achieve optimal signal-to-noise ratios and reproducibility. Hoechst 33342 stained the nuclei of all cells, producing a highly fluorescent blue signal, detectable using a standard DAPI filter (excitation: 380/30 nm, emission: 450/50 nm). Sytox selectively stained the nuclei of cells with compromised plasma membranes, producing a highly fluorescent green signal, detectable with a standard FITC filter (excitation: 470/40 nm, emission: 525/50 nm). Mitosox stained the mitochondria in live cells, producing a red fluorescent signal, detectable with the Texas red filter (excitation: 560/40 nm, emission 630/75 nm).

Step 3: Taking well plate 1 out from the biosafety cabinet and putting it back into the incubator at 37 °C for 30 min for staining to take place.

Step 4: Taking well plate 1 out from the incubator and putting it on the top of the heat block (2052FS Dual Dry Bath Incubator, Fisher Scientific) for 10 min at 37 °C. The heat block was pre-heated and verified for consistent temperature across its surface using a calibrated thermometer prior to each experiment. The well plate remained on the heat block for 10 min, ensuring that each well experienced the same controlled temperature.

Step 5: Taking well plate 1 out from the heat block and putting it in the biosafety cabinet.

Step 6: In the biosafety cabinet, using a micropipette, removing the staining reagent solution from the 22 wells of well plate 1. Then, using a micropipette, 100 µL of 1xPBS solution was added to each of the 22 wells, and afterwards, the PBS solution was removed from the 22 wells. This step was repeated twice to remove residual staining reagent properly from the 22 wells.

Step 7: Evaluating cells’ responses for well plate 1 using the plate reader (SpectraMax M3, Molecular devices) and the microscope (Echo revolution fluorescence microscope, model: RON-K, BICO company). The plate reader data were stored on the computer in text format. Microscopic images were captured of five randomly selected samples from well plate 1. Image views were selected randomly by alternating sample locations to avoid any pattern or bias. Within each sample, fields of view were chosen to capture regions with evenly distributed cells, ensuring consistency in analysis. The image files were stored on a computer in the TIFF (.tif) format.

The text files of the plate reader data were transferred into an Excel file and then the data were analyzed using the OriginPro software (version 2024b). The unit of the plate reader data was relative fluorescence units (RFU). The TIFF (.tif) files of the microscope images were analyzed using ImageJ Fiji software (version 1.54f). The number of all cells (*t_i_*) and the number of dead cells (*d_i_*) in each of the images (*i* = 1 to 5) of a particular well plate were counted. *V_c_* is the average cell viability (%) for a particular well plate and was calculated using Equation (1).

(1) $$V_{c}=\frac{\sum_{i=1}^{5}\left(\frac{t_{i}-d_{i}}{t_{i}}\times 100\right)}{5}$$

### 2.6. Statistical Analysis

Statistical analysis was conducted using OriginPro software (version 2024b). Initially, the normality test (Shapiro–Wilk test) was used to assess the normality of the data distribution. For the cell viability data that passed the normality test, a one-way ANOVA was performed. For the cell viability data that failed the normality test, a nonparametric Kruskal–Wallis ANOVA was performed. Furthermore, a mean pair-comparison test was used to analyze differences in experimental data between the three experimental conditions.

## 3. Results and Discussion

Experimental data for Hoechst 33342 intensity, Sytox intensity, Mitosox intensity, and cell viability are presented in Table 2. Table 2 shows the relative fluorescence unit (RFU) values for Hoechst 33342, Sytox, and Mitosox assays at three levels of temperature. RFU represents the intensity of fluorescence, in arbitrary units, emitted by the sample and detected by the plate reader’s photomultiplier tube, correlating with the levels of DNA content, cell death, and mitochondrial ROS. The nonparametric Kruskal–Wallis ANOVA was utilized to determine the statistical significance of the effects of the temperature on Hoechst 33342 intensity, Sytox intensity, and Mitosox intensity, as these data failed the normality test. To evaluate the statistical significance of the effects of temperature for the cell viability, a one-way ANOVA was performed on the experimental data, as these data passed the normality test. Table 3 presents the p-values and the effect sizes for the effects of temperature on Hoechst 33342 intensity, Sytox intensity, Mitosox intensity, and cell viability. The effect size estimate used here is the coefficient *η*^2^. To calculate *η*^2^ for the ANOVA test, the following Equation (2) was used [18]:

(2) $$\eta^{2}=\frac{{\sum of\;squares}_{effect}}{{\sum of\;squares}_{total}}$$

For the Kruskal–Wallis ANOVA test, the effect size *η*^2^ was calculated using the following Equation (3) [18]. *H* is the value obtained in the Kruskal–Wallis test (chi-squared), *k* is the number of groups (three levels of temperature), and n is the total number of observations.

(3) $$\eta_{H}^{2}=\frac{H-k+1}{n-k}$$

Table 4 presents the *p*-values from mean pair-comparison test for the effects of temperature on Hoechst 33342 intensity, Sytox intensity, Mitosox intensity, and cell viability.

**Figure 5:**
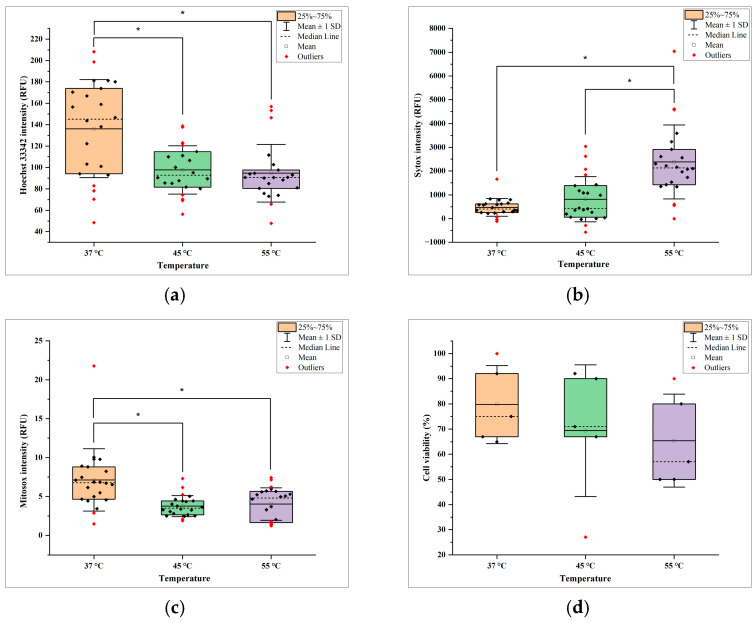
Effects of temperature on (**a**) Hoechst 33342 intensity, (**b**) Sytox intensity, (**c**) Mitosox intensity, and (**d**) cell viability (* indicates *p*-value < 0.05).

The effects of temperature on Hoechst 33342 intensity, Sytox intensity, Mitosox intensity, and cell viability are shown in Figure 5a, Figure 5b, Figure 5c, and Figure 5d, respectively. The error bars in these figures represent the standard deviation among the samples at one level of temperature. Figure 5a shows that Hoechst 33342 intensity (an indicator of all cells, stained blue) was the highest at 37 °C and the lowest at 55 °C. The reduced intensity at 55 °C reflects severe membrane damage and nuclear integrity loss. This trend aligns with that in a reported study on human mesenchymal stem cells exposed to elevated temperatures [14]. In that study, cell viability and metabolism were maintained when being exposed to up to 48 °C for 150 s, but significant cell death was observed when being exposed to 58 °C for 45 s [14]. Figure 5b shows that increasing the temperature from 37 °C to 55 °C significantly increased Sytox intensity (dead cell indicator by green stain), reflecting a higher degree of cell death. The greatest increase in Sytox intensity occurred at 55 °C, indicating a substantial increase in dead cells.

Figure 5c shows that Mitosox intensity (mitochondrial superoxide indicator by red stain) of the samples exposed to temperature of 37 °C was higher than those exposed to temperature of 45 °C and 55 °C. This could be due to the presence of a larger population of live cells capable of undergoing oxidative stress, which Mitosox specifically detects. In the samples exposed to temperature of 37 °C, cells were metabolically active, and normal mitochondrial function were able generate a baseline level of reactive oxygen species (ROS). Thus, even in the samples exposed to high temperature, the active mitochondria in the live cells were able to produce detectable ROS, leading to higher Mitosox intensity. In the samples exposed to a temperature of 45 °C, Sytox intensity increased, leading to fewer metabolically active cells capable of generating mitochondrial ROS. As a result, the overall Mitosox intensity decreased due to the loss of these cells. In samples exposed to 55 °C, Sytox intensity was higher (indicating more cell death) and Mitosox intensity was elevated (indicating that live cells exhibited extreme stress). However, due to the reduced number of viable ROS-producing cells, overall Mitosox intensity remained lower than in samples exposed to 37 °C. Thus, the trend in Mitosox intensity reflects a balance between the number of live cells and their mitochondrial activity in the samples exposed to different levels of temperature. Figure 5d shows that cell viability in the samples exposed to the temperature of 37 °C was higher than in the samples exposed to the temperatures of 45 °C and 55 °C.

**Figure 6:**
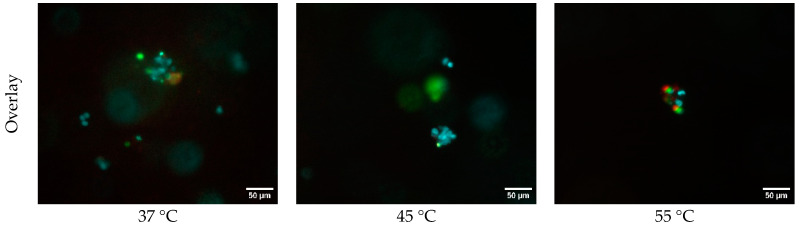

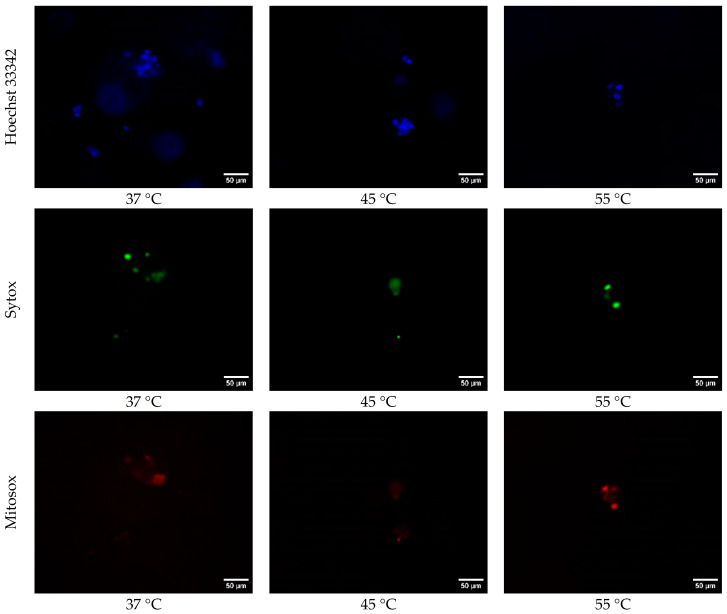
Fluorescence microscopy images of all cells (blue, Hoescht 33342), dead cells (green, Sytox), and cells under mitochondrial stress (red, Mitosox) in 3D printed samples exposed to temperatures of 37 °C, 45 °C, and 55 °C.

Figure 6 shows fluorescence microscopy images of samples exposed to temperatures of 37 °C, 45 °C, and 55 °C, stained with Hoechst 33342 (blue) for all cells, Sytox (green) for dead cells, and Mitosox (red) for mitochondrial oxidative stress. For the samples exposed to a temperature of 37 °C, the overlay image demonstrates a healthy distribution of cells, as indicated by the prominent blue Hoechst 33342 staining, with some red Mitosox signal indicating low levels of mitochondrial oxidative stress. The Sytox signal in green is sparsely present, indicating minimal cell death at this temperature. Clusters of cells in the images represent cells proliferated in the samples after incubating for three days post printing. The Hoechst 33342 images in Figure 6 show that the number of all cells was noticeably higher in the samples exposed to a temperature of 37 °C than those that were exposed to temperatures of 45 °C and 55 °C. From the Mitosox images in Figure 6, it can be observed that cells in the samples exposed to temperature of 55 °C experienced mitochondrial oxidative stress. This means that human HBE cells compromised their metabolic activity when being exposed to higher temperature. This is likely because elevated temperatures, particularly at 55 °C, cause cellular damage by disrupting mitochondrial function, leading to increased production of ROS. The accumulation of ROS induces oxidative stress, which impairs the cell’s metabolic activity and can lead to cell death. In other words, at higher levels of temperature, thermal stress overwhelms the cells’ ability to maintain homeostasis, resulting in compromised mitochondrial activity and, consequently, reduced cell proliferation and viability.

## 4. Conclusions

This study addresses a knowledge gap in the literature regarding how high temperature affects human bronchial epithelial (HBE) cells. In this study, three sets of samples were prepared using 3D printing of cell-laden droplets. These samples were exposed to three levels of temperature (37 °C, 45 °C, and 55 °C). Cells’ responses, specifically cell viability and oxidative stress of human HBE cells in these samples were evaluated. In the samples exposed to 45 °C and 55 °C, cell viability was compromised, and mitochondrial oxidative stress was observed at comparable levels, raising intriguing questions about the underlying mechanisms. Further analysis of these cellular mechanisms will be conducted in a future study, where additional assays targeting apoptotic and necrotic cell markers will be employed. These assays are crucial to distinguish between cell death pathways and residual oxidative processes that may occur at extreme temperatures. Apoptosis-specific markers (such as caspase activity assays) alongside necrotic indicators will be used to confirm whether the residual Mitosox signal at 55 °C is due to ongoing oxidative stress in dying cells or due to altered respiration. To examine if altered Mitosox red signal is due to altered respiration, future studies will employ assessments of oxygen consumption and extracellular acidification rates in the face of temperature stress. Beyond metabolic stress, we will seek to examine shifts in the unfolded protein response and molecular responses, such as DNA replication, in the future. These approaches will provide further understanding regarding cellular responses. We will seek to also include more long-term heat exposure experiments, in which cell-laden constructs are exposed for more than 10 min, as well as oscillatory exposures, in which cells are exposed to heat stress for a short time, then returned to physiological temperatures, before returning to the stressful temperatures.

This study demonstrates how 3D printed samples containing human bronchial epithelial cells can serve as valuable tools for assessing cellular responses to extreme temperatures, with potential applications in occupational health monitoring. By further developing this technique, multi-layered 3D printed constructs could be designed to simulate high-heat exposure environments (for different levels of exposure time), such as those encountered by pilots, firefighters, and industrial workers. Additionally, these constructs could serve as drug testing platforms to evaluate compounds aimed at reducing thermal-induced cellular damage.

## Figures and Tables

**Table 1:** Several reported studies on the effects of extreme temperature.

Extreme Temperature	Test Model	Reference
38 °C within fighter cockpit for 15 min	Human (male)	[5]
75.7 ± 0.86 °C in a sauna for 15 min	Human (male and female)	[12]
37–44 °C in a thermostat bath for 20 min	Mouse neural stem cells	[13]
38 °C, 48 °C, and 58 °C; heated medium poured into wells with cells for durations of 45, 80, and 150 s	Human mesenchymal stem cells	[14]
40 ± 0.5 °C in an incubator for 1 h	Male Wistar rats’ lung tissue	[15]
42 °C using a heat block for 5 h	Tardigrade species (Paramacrobiotus experimentalis)	[16]

**Table 2:** Experimental data for three levels of temperature.

	Hoechst 33342 Intensity (RFU)		Sytox Intensity (RFU)		Mitosox Intensity (RFU)		Cell Viability (%)	
	Mean	Standard Deviation	Mean	Standard Deviation	Mean	Standard Deviation	Mean	Standard Deviation
37 °C	136.24	45.82	474.78	371.54	7.13	4.01	79.8	15.51
45 °C	97.73	22.57	815.42	948.28	3.76	1.35	69.4	26.18
55 °C	94.66	26.93	2385.11	1556.99	4.02	2.09	65.4	18.46

**Table 3:** *p*-values and effect sizes for the effects of temperature on Hoechst 33342 intensity, Sytox intensity, Mitosox intensity, and cell viability.

	Hoechst 33342 Intensity	Sytox Intensity	Mitosox Intensity	Cell Viability
*p*-value	0.0011	0.0001	0.0003	0.537
Effect size	0.1594	0.3613	0.2293	0.0983

**Table 4:** *p*-values from mean pair-comparison test for the effects of temperature on Hoechst 33342 intensity, Sytox intensity, Mitosox intensity, and cell viability.

Pair Comparison	Hoechst 33342 Intensity	Sytox Intensity	Mitosox Intensity	Cell Viability
37 °C, 45 °C	0.0088	1	0.0005	0.70995
37 °C, 55 °C	0.00191	0.0001	0.00441	0.52746
45 °C, 55 °C	1	0.0004	1	0.94934

## Data Availability

The authors confirm that the data to support the findings of this study are available within the article or upon request to the corresponding author.
